# Effect of Increasing or Decreasing Use of Polypharmacy on Recovery of Activities of Daily Living in Patients With Stroke in the Recovery-Phase Rehabilitation Ward: A Retrospective Cohort Study Using Propensity Score Matching

**DOI:** 10.1155/srat/2381790

**Published:** 2024-12-13

**Authors:** Shunsuke Hanaoka, Kaede Iwabuchi, Toshiyuki Hirai, Toshiichi Seki, Hiroyuki Hayashi

**Affiliations:** ^1^Laboratory of Pharmacotherapy, School of Pharmacy, Nihon University, 7-7-1 Narashinodai, Funabashi-shi, Chiba 274-8555, Japan; ^2^Department of Pharmacy, Hitachi, Ltd. Hitachinaka General Hospital, 20-1, Ishikawa-cho, Hitachinaka-shi, Ibaraki 312-0057, Japan

## Abstract

**Background:** Polypharmacy is a predictor of adverse outcomes, making its management crucial for improving patient health and recovery. Managing polypharmacy is particularly challenging in patients with stroke with many comorbidities and sequelae. Although reducing inappropriate prescribing is necessary, the number of medications may increase to effectively implement secondary prevention, potentially offsetting any changes in medication count. For patients with stroke undergoing recovery-phase rehabilitation, balancing secondary prevention and optimizing drug use early without hindering recovery of activities of daily living are crucial. This study is aimed at examining the effect of increasing or decreasing the use of polypharmacy on recovery of motor and cognitive function during recovery-phase rehabilitation in patients with stroke.

**Methods:** The study was conducted from July 2010 to June 2019 among patients with stroke discharged from the convalescent rehabilitation ward during the study period. Patients who were using more than five drugs on admission and had either an increase or decrease in the number of drugs used on discharge were compared. Propensity score matching (PSM) was used to control for background variables such as patient demographics, laboratory values, and functional independence measure (FIM) scores at baseline. The primary outcomes were motor, cognition, and total FIM gain.

**Results:** Of the 226 patients initially enrolled, 156 were matched on propensity score. The total motor FIM gain, total cognitive FIM gain, and total FIM gain were significantly higher in the decreased group than in the increased group (*p* = 0.0139, *p* = 0.0377, and *p* = 0.0077, respectively).

**Conclusion**: In patients with stroke, reducing rather than increasing the number of drugs administered during recovery-phase rehabilitation could improve rehabilitation outcomes. Therefore, it is important to consider whether the drugs are essential for the patient and proactively revise the drug regimen to ensure rapid rehabilitation of patients with stroke.

## 1. Introduction

In poststroke patients, symptomatic treatments for sequelae, such as poststroke depression [1–5], poststroke sleep disorders [6–8], spasticity [9, 10], central pain [11–15], lower urinary tract dysfunction [16], and epilepsy [17–21], may be required. Overlapping these treatments without clear goals can lead to polypharmacy, which poses risks of decreased motor and cognitive functions, decreased liver and kidney function, and adverse drug interactions. Previous reports have identified polypharmacy as a predictor of adverse outcomes [22, 23].

Immediate and sustained implementation of effective secondary prevention strategies is needed for patients with stroke [24]. Lifestyle-related diseases such as hypertension, diabetes, dyslipidemia, and hypercholesterolemia increase the risk of stroke [25]. After a stroke onset, therapeutic drugs are often supplemented with medications for these diseases to prevent recurrence. Several studies have assessed the stroke recurrence rate [24, 26–28]. According to one study, the recurrence rate decreased until 2005 and then stabilized, possibly due to unsuccessful secondary prevention in some patients [26]. Ensuring treatment for these groups could have a positive effect on the outcomes. Additionally, early initiation of secondary prevention strategies in the hospital may improve patient adherence and success [24].

Although inappropriate prescribing needs to be reduced, the number of drugs may increase to effectively implement secondary prevention, potentially offsetting any changes in the number of medications. However, managing polypharmacy is challenging for patients with multiple comorbidities and stroke sequelae.

Sequelae of cerebrovascular disease can cause a decline in physical function and activities of daily living (ADLs). Thus, rehabilitation should be planned according to the pathological condition within 24–48 h after stroke onset [29, 30] and rehabilitation should be started early during the convalescent period in a multidisciplinary setting, and prioritizing safety is essential to prevent a decline in ADLs due to these sequelae [31, 32]. The convalescent period represents the subacute recovery phase. Convalescent rehabilitation wards, consisting of a group of beds for patients receiving comprehensive rehabilitation care, were established in Japan based on cooperation among multiple professions [33].

Immediate and sustained implementation of effective secondary prevention strategies without interfering with ADL recovery is crucial for patients with stroke undergoing convalescent rehabilitation. If reducing the number of medications used does not hinder ADL recovery through rehabilitation, it is important to balance secondary prevention and optimal drug use early in the course of rehabilitation. However, to our knowledge, no previous studies have assessed how changes in polypharmacy, considering both secondary use medications and those used for poststroke sequelae, affect rehabilitation outcomes in patients with stroke.

In inpatient rehabilitation, tracking patients' functional progress is crucial [34]. Treatment effectiveness can be measured using either performance-based measures (PBMs) or patient-reported outcome measures (PROMs). PBMs refer to objective, quantifiable data collected to assess treatment effectiveness. These structured observational tests are an accurate measure of function [35]. PROMs measure the subjective elements of patients' conditions, including health-related quality of life, pain intensity, activity limitations, participation restrictions, satisfaction, and adherence to treatment [36, 37]. PROMs provide evidence of the effect of interventions on patient symptoms and quality of life. However, being subjective, they are prone to bias. Therefore, PROMs should be used alongside objective outcomes, such as PBMs, especially when blinded assessment by clinicians or researchers is not possible [37].

In this study, the functional independence measure (FIM), widely used as an objective index in rehabilitation, was used to evaluate ADLs. Unlike the EuroQol five dimensions (EQ-5D) and the Medical Outcomes Study 36-Item Short-Form Health Survey (SF-36), FIM does not cover all aspects of the PROMs recommended by the 2021 Academy of Neurologic Physical Therapy (ANPT) Stroke Evidence Database to Guide Effectiveness II (StrokEDGE II). It also does not include all recommended PBMs such as the 10-meter walk test and the 6-minute walk test. However, StrokEDGE II endorses the FIM as an outcome measure for patients in stroke inpatient rehabilitation [38, 39]. Additionally, in Japan, to determine the fee for inpatient care in a convalescent-stage rehabilitation ward, it is necessary to regularly measure the FIM. The motor FIM scores at the time of admission and discharge are required to clarify the effectiveness of rehabilitation when creating a rehabilitation implementation plan.

Therefore, this study is aimed at investigating the effect of an increase or decrease in the number of drugs used in patients with polypharmacy recovering from stroke in a convalescent rehabilitation ward. Additionally, we investigated the use of medications for secondary prevention, chronic diseases, and poststroke sequelae. We hypothesized that patients in whom the number of drugs used decreased would have better rehabilitation outcomes than those in whom the number of drugs used increased.

## 2. Materials and Methods

### 2.1. Study Design

This was a single-center retrospective observational cohort study. This study complied with the standards of the Declaration of Helsinki and the current Japanese ethical guidelines. The study protocol was approved by the Nihon University School of Pharmacy Ethics Committee (Approval Number 23-001) which approved the use of opt-out consent owing to the retrospective study design.

### 2.2. Study Population

This study included 936 patients with stroke discharged from the convalescent rehabilitation ward of the Hitachinaka General Hospital between July 2010 and June 2019. Patients who had a stroke or had undergone surgery within the previous 2 months were admitted to the convalescent rehabilitation ward. Intensive rehabilitation focused on improving ADLs such as eating, dressing, toileting, bathing, mobility, and communication was conducted to prevent bedridden states and facilitate the return to home and society. Patients with missing baseline characteristic values on admission (serum albumin (Alb), alanine aminotransferase (ALT), estimated glomerular filtration rate (eGFR), body mass index (BMI), hemoglobin (Hb), and total FIM score) were excluded. The remaining patients were included in the analysis.

### 2.3. Convalescent Rehabilitation Ward

In Japan, convalescent rehabilitation wards are similar to inpatient rehabilitation facilities in North America and Europe and are the main inpatient rehabilitation facility covered by the Ministry of Health, Labor and Welfare of Japan medical insurance system. The convalescent period represents the subacute recovery phase. All patients with stroke and those with other neurological diseases with severe disability and cognitive disorders, orthopedic diseases, and disuse/myopathy syndrome who require assistance in regaining ADLs after receiving treatment in acute hospitals are transferred to the convalescent rehabilitation ward [40].

### 2.4. Outcome Evaluation Criteria

The primary outcomes were motor and cognition FIM gain total and total FIM gain. The FIM consists of 18 items, including 13 motor ADL items (motor FIM) and five cognition ADL items (cognition FIM). Motor FIM includes self-care (eating, grooming, bathing, upper dressing, lower dressing, and toileting), sphincter control (bladder and bowel management), mobility (bed/chair/wheelchair, toilet, and tub/shower transfers), and locomotion (walking/wheelchair and stairs). Cognition FIM comprises communication (comprehension and expression) and social cognition (social interaction, problem solving, and memory).

Each FIM item was evaluated using a seven-point Likert scale ranging from total assistance (1 point) to complete independence (7 points). The total score (total FIM) ranged from 18 to 126 points. The care team consisted of doctors in convalescent wards, nurses with a long work history in the ward, rehabilitation staff (physical, occupational, and speech-hearing therapists), and pharmacists who discussed and scored the FIM. The FIM gains of each item, a measure of ADL improvement during admission to the ward, were used as the outcome of this study. FIM gain was calculated by subtracting the FIM score on admission from the FIM score on discharge from the recovery unit.

### 2.5. Variables

We retrospectively extracted information on admission and discharge and during the stay in the rehabilitation ward from electronic medical records. The data included patient characteristics (sex, age, BMI, and length of stay), laboratory results (ALT, eGFR, Alb, and Hb), medical history (hypertension, Type 2 diabetes mellitus, heart disease, atrial fibrillation, dyslipidemia, hypercholesterolemia, cerebral infarction, intracerebral hemorrhage, and subarachnoid hemorrhage), and clinical events during the stay (falls). Data on drug use (number of drugs used, polypharmacy, and anticholinergic cognitive burden (ACB) score) and specific drugs used during the stay (antiplatelet agents, anticoagulants, antihypertensive drugs, antidiabetic agents, statins, benzodiazepines (BDZs), selective serotonin reuptake inhibitors (SSRIs), and tricyclic antidepressants (TCAs)) were also collected. Additionally, FIM total scores were recorded, including individual item scores on admission and discharge, and the FIM total gain.

Age was defined as the age on admission to the ward. In addition to comparing age (years), age was categorized as under 65 years (< 65 years) and 65 years and older (≥ 65 years) according to the World Health Organization guidelines. The length of convalescent rehabilitation stay was calculated by subtracting the admission date from the discharge date. In addition to comparing BMI (kilograms per square meter) on admission and discharge, a low BMI was defined as ≤ 20 kg/m^2^, as this value is associated with a statistically higher risk of total mortality and the need for nursing care insurance services in Japan [41, 42].

ALT and Hb on admission and during the stay were categorized according to Japanese reference intervals of the Japanese Committee for Clinical Laboratory Standards. The criteria are as follows: high ALT: > 42 IU/L (male), > 23 IU/L (female); low Hb: < 13.7 g/dL (male), and < 11.6 g/dL (female) [43]. The criterion for Alb was based on previous reports, with Alb < 3.5 g/dL indicating hypoalbuminemia [44]. The criterion for eGFR was eGFR < 60 mL/min/1.73 m^2^, corresponding to chronic kidney disease (CKD) Class G3a or lower [45].

The data on medical history included diseases associated with an increased risk of stroke and the stroke subtype. Heart disease included major conditions such as angina pectoris, myocardial infarction, heart failure, heart valve disease, and myocarditis. Atrial fibrillation was recorded separately. In addition to recording stroke subtypes, including cerebral infarction, intracerebral hemorrhage, and subarachnoid hemorrhage, as the primary cause for requiring rehabilitation, past occurrences were recorded.

The number of prescribed drugs on admission and discharge was defined as the oral medications prescribed during the stay. Drugs prescribed in other hospitals were not counted. Additionally, the number of prescribed drugs was classified into two groups: < 5 drugs (no polypharmacy group) and ≥ 5 drugs (polypharmacy group), with the ≥ 5 group considered to represent polypharmacy based on a previous report [46].

The ACB score on admission and discharge was calculated based on the ACB scale 2012 update. Criteria for categorization were as follows: score of 1: evidence from in vitro data that the chemical entity has antagonist activity at the muscarinic receptor; score of 2: evidence from literature, prescriber's information, or expert opinion of a clinical anticholinergic effect; and score of 3: evidence from literature, expert opinion, or prescriber's information that the medication may cause delirium [47]. The total ACB score was calculated by summing the scores of all drugs the patient was taking on admission and discharge. For example, if a patient was taking two drugs with an ACB score of 1 and one drug with an ACB score of 3, the total ACB score would be (1 × 2) + (3 × 1) = 5. We also investigated the number of patients using drugs with the highest ACB score of 3.

To understand the medications used by patients, we counted specific types of drugs administered during their stay. For stroke secondary prevention, we counted patients using antiplatelet agents (including aspirin and P2Y_12_ inhibitors) and anticoagulants (including warfarin and direct oral anticoagulants). For secondary stroke prevention, we counted patients using antihypertensive drugs, antidiabetic agents, and statins. Antihypertensive drugs included thiazides, calcium channel blockers, angiotensin-converting enzyme inhibitors, and angiotensin II receptor blockers. Antidiabetic agents included biguanides, thiazolidinediones, sulfonylureas, glinides, alpha-glucosidase inhibitors, dipeptidyl peptidase-4 inhibitors, and sodium-glucose cotransporter-2 inhibitors. For symptomatic treatment of stroke sequelae, we assessed BDZ, TCA, and SSRI use.

We recorded the FIM total scores on admission and discharge, as well as the FIM gain. In addition to comparing FIM total scores, FIM gain scores were categorized and compared according to whether there was a minimally clinically important difference (MCID), based on a previous report [48] included in the StrokEDGE II compendium: motor FIM total ≥ 17, cognitive FIM total ≥ 3, and total FIM ≥ 22. The primary outcomes of this study were the total motor FIM, total cognition FIM, and total FIM gain scores.

### 2.6. Statistical Analysis

The analysis was performed using JMP Pro 17 (SAS Institute Inc., Cary, North Carolina, United States). Patient characteristics, laboratory values, medical history, clinical events, drug use status, FIM on admission and discharge, and FIM gain between groups were compared. Welch's *t*-tests were used to compare continuous variables, whereas chi-square tests were used for categorical variables. Continuous variables were reported as the mean and standard deviation (SD), and categorical variables were reported as frequencies and percentages (%).

First, to provide the clinical and demographic information of the whole study cohort, we compared baseline patient characteristics and FIM scores between two groups: patients prescribed less than five drugs on admission (no polypharmacy-on-admission group) and patients prescribed five or more drugs on admission (polypharmacy-on-admission group).

Second, within the no polypharmacy-on-admission group, we compared three subgroups: decreased, increased, and no change in drug use. The decreased group included patients with fewer medications on discharge than on admission, the increased group comprised patients with more medications on discharge than on admission, and the no change group consisted of patients whose number of medications on admission and discharge remained the same.

Third, based on the comparison of the three groups, we excluded the no change group, which had the highest FIM values on both admission and discharge. To investigate the effect of medication changes during rehabilitation while controlling for confounders, we performed propensity score matching (PSM) on the increased and decreased drug use groups. The patients who were included after matching were the main target of the study. After adjusting for the effects other variables on FIM gain, we ensured balance between the two groups.

The variables used to calculate the propensity score (PS) were selected based on medical knowledge and previous reports [49–57]: cerebral infarction (number of patients), heart disease (number of patients), Type 2 diabetes mellitus (number of patients), BMI < 20.0 kg/m^2^ on admission (number of patients), Alb < 3.5 g/dL on admission (number of patients), low Hb on admission (number of patients), age, number of drugs used on admission, and total FIM on admission. The PS was calculated by including the selected variables in a multivariable logistic regression model. The appropriateness of the PS calculation was verified using *c*-statistics. Generally, a *c*-statistic of ≥ 0.6 is desirable. Based on the calculated PS, matching was performed with decreased and increased drug use to adjust for the influence of confounding variables. Nearest-neighbor matching was used as the matching method, and the threshold (caliper) indicating the distance between PS values to be matched was set to 0.2 × SD of the logit-transformed PS values [58]. The matching balance was evaluated based on whether the standardized difference (Std. Dif.) was < 0.1. A Std.Dif.<0.1 indicated that the groups were appropriately matched [58]. These procedures made this quasiexperimental study approximate a randomized controlled trial, even though it was retrospective.

After PSM, the primary endpoints of total motor FIM gain, total cognition FIM gain, and total FIM gain were compared using the Mann–Whitney *U*-test, in addition to Welch's *t*-test. Statistical significance was set at *p* < 0.05.

Additionally, to evaluate the effect of reducing the number of drugs from five or more to fewer than five, we compared two subgroups within the decreased group before PSM: those receiving five or more drugs on discharge (polypharmacy on discharge) and those receiving fewer than five drugs on discharge (no polypharmacy on discharge). We analyzed patient information on admission (demographics and medical history) and FIM gain variables (total motor FIM gain, total cognition FIM gain, and total FIM gain).

## 3. Results

### 3.1. Study Population

**Figure 1:**
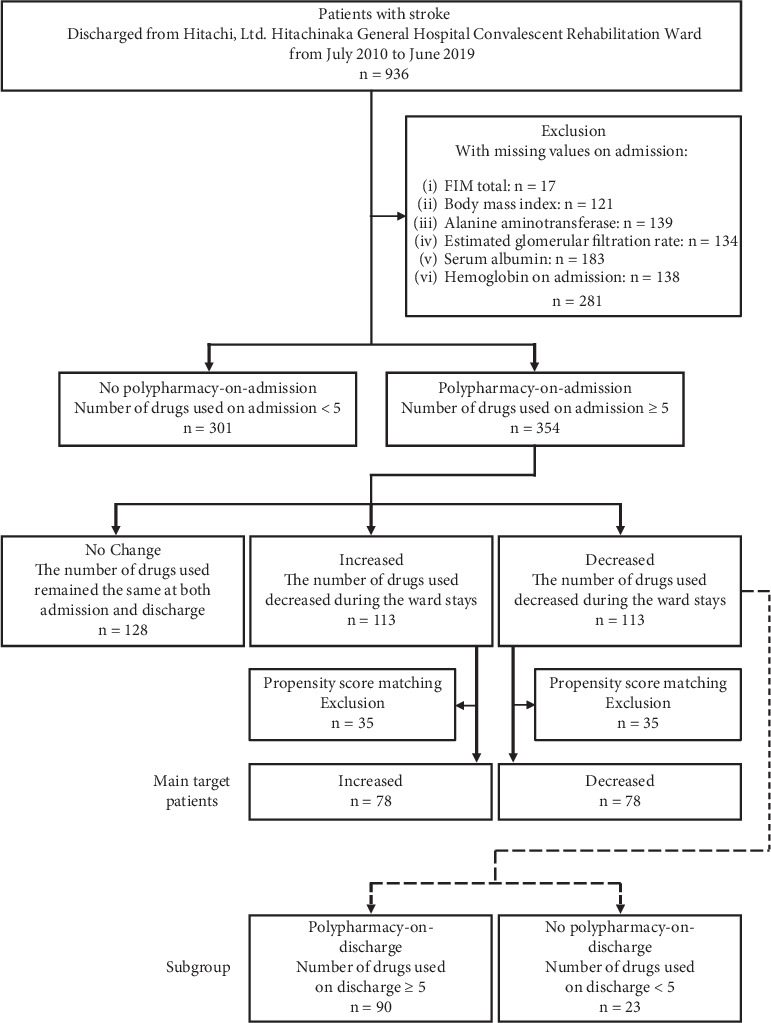
Flowchart showing the selection of patients for the analysis and the subgroups that were compared.

Figure 1 shows the study population. Of 936 patients with stroke, 281 were excluded owing to missing data (total FIM, BMI, ALT, eGFR, Alb, and Hb on admission), leaving 655 patients available for analysis. These 655 patients were divided into two groups based on the presence of polypharmacy on admission: 301 patients in the no polypharmacy-on-admission group and 354 patients in the polypharmacy-on-admission group.

The polypharmacy-on-admission group was divided into three subgroups based on the change in the number of drugs prescribed between admission and discharge: no change (128 patients), increased (113 patients), and decreased (113 patients). The no change group had more stable ADL conditions on admission and discharge than the other two groups. Therefore, the no change group was excluded from PSM, and the remaining 226 patients (increased and decreased drug use groups) were matched using PSM. After PSM, 78 patients in the increased group were matched and compared with 78 patients in the decreased group. These 156 patients were the main target of the analysis.

The decreased drug use group was divided into two groups based on whether they had polypharmacy on discharge: polypharmacy on discharge (90 patients) and no polypharmacy on discharge (23 patients).

### 3.2. Comparison of Baseline Patient Information on Admission Between the No Polypharmacy- and Polypharmacy-on-Admission Groups

The information on admission and FIM total scores for 665 patients were compared between two groups: the no polypharmacy-on-admission group and the polypharmacy-on-admission group (Tables 1 and 2). The polypharmacy-on-admission group was significantly older (*p* = 0.0007) and had higher ACB scores (*p* < 0.0001) than the no polypharmacy-on-admission group. Additionally, the polypharmacy-on-admission group had a significantly higher proportion of patients using drugs with an ACB score of 3 on admission. The polypharmacy-on-admission group also had a significantly higher proportion of patients with reduced kidney function (*p* = 0.0001) and lower Hb levels (*p* = 0.0003).

In terms of medical history, the polypharmacy-on-admission group had a significantly higher proportion of patients with Type 2 diabetes mellitus (*p* < 0.0001), heart disease (*p* = 0.0004), and cerebral infarction (*p* < 0.0001) and a significantly lower proportion of patients with intracerebral hemorrhage. Although the proportion of patients with hypertension was higher in the polypharmacy-on-admission group the difference in the prevalence of hypertension between groups was not statistically significant.

The polypharmacy-on-admission group had significantly higher proportion of patients using antiplatelet agents (*p* < 0.0001), statins (*p* < 0.0001), antidiabetic agents (*p* < 0.0001), antihypertensive drugs (*p* = 0.0010), and BDZs (*p* = 0.0002).

There were no significant differences in FIM values between the two groups on admission and discharge. However, the polypharmacy-on-admission group had significantly lower gains in total motor FIM (*p* = 0.0100) and total FIM (*p* = 0.0087). Although the proportion of patients with MCID values for gains in total motor FIM, total cognition FIM, and total FIM was slightly higher in the no polypharmacy-on-admission group, the differences in MCID values did not differ significantly between the two groups.

In summary, the patients in the polypharmacy-on-admission group were older with higher ACB scores and more chronic medical conditions; were taking more drugs for treating these medical conditions; and had lower kidney function, Hb levels, and lower total FIM gains, particularly total motor FIM and total FIM gains than those in the no polypharmacy-on-admission group.

### 3.3. Comparison of the Patient Characteristics and FIM Total Scores Between the No Change, Increased, and Decreased Drug Use Groups

We compared patient information and FIM scores on admission, discharge, and during the stay between the no change, increased, and decreased drug use groups before PSM. The results are shown in Tables 3, 4, and 5. Compared with the other two groups on admission, the no change group was younger (*p* = 0.0256) with a lower proportion of patients aged 65 years or older (*p* = 0.0003), used fewer drugs on admission (*p* = 0.0016), and was less likely to have heart disease (*p* = 0.0159), whereas the decreased drug use group was less likely to have cerebral infarction (Table 3).

Compared with the other three groups on discharge, the decreased drug use group were prescribed fewer medications (*p* < 0.0001), had lower ACB scores (*p* = 0.0057), and had a lower proportion of patients using ACB score 3 drugs (*p* = 0.0030). During the ward stay, the no change group had fewer patients with low Hb and fewer patients using BDZs (Table 4).

Regarding the FIM scores, the no change group had the highest total motor FIM and total FIM scores both on admission and on discharge. However, the total FIM gains did not differ significantly between the three groups (Table 5). In summary, the no change group was younger, had fewer chronic medical conditions, and exhibited a higher level of independent ADL both on admission and on discharge than the other two groups.

### 3.4. Adjustment of Covariates by PSM

The distribution of the variables before and after PSM is shown in Tables 6 and 7. Based on the comparison of the results among the no change, increased, and decreased drug use groups, we included 226 patients (113 decreased and 113 increased) in the PSM analysis, excluding the no change group. The no change group had stable ADL conditions at both admission and discharge compared with the other two groups. The *c*-statistic for the PS calculation from the selected covariates was 0.709. PSM showed that 69.9% of the patients in the increased drug use group were matched with patients in the decreased drug use group. After PSM, the Std. Dif. for each covariate was below 0.1, indicating that the covariate distributions in the two groups were sufficiently balanced. After PSM, 156 patients (78 increased and 78 decreased) were included in the analysis, comprising 68 women and 88 men. Their mean age was 74.0 ± 11.4 years, and the mean length of stay was 72.5 ± 36.1 days.

### 3.5. Comparison of Patient Characteristics on Admission After PSM

The patient characteristics, laboratory abnormalities, and medical history on admission in the increased and decreased drug use groups after PSM are shown in Table 8. None of the variables differed significantly between the two groups.

### 3.6. Comparison of Patient Characteristics on Discharge and During the Stay in the Increased and Decreased Drug Use Groups After PSM

The patient characteristics on discharge, laboratory abnormalities, and clinical events during hospitalization in the increased and decreased drug use groups after PSM are shown in Table 9. None of the variables differed significantly between the two groups.

### 3.7. Comparison of Drug Use Status on Admission and Discharge and During the Ward Stay After PSM

The drug use status on admission, on discharge, and during the ward stay after PSM is shown in Table 10. The number of drugs used on discharge (*p* < 0.0001), the proportion of patients with polypharmacy on discharge (*p* < 0.0001), and the ACB score on discharge (*p* = 0.0004) were significantly lower in the decreased drug use group than in the increased drug use group. Additionally, patients in the decreased group were significantly less likely to be using ACB score 3 drugs on admission (*p* = 0.0498) and discharge (*p* = 0.0004). The other variables did not differ significantly between the two groups.

### 3.8. Comparison of the Total FIM Scores After PSM

The total FIM scores on admission and discharge and the FIM gain in the decreased and increased groups after PSM are shown in Table 11. The total FIM scores on admission and discharge did not differ significantly between the two groups.

In terms of FIM gain, the total motor FIM (*p* = 0.0139), total cognition FIM (*p* = 0.0377), and total FIM (*p* = 0.0077) were significantly higher in the decreased drug use group than in the increased drug use group. Although the number of patients with MCID values for gains in the motor FIM, cognition FIM, and total FIM was slightly higher in the decreased group, the MCID values did not differ significantly between the two groups.

Similar findings were observed for the total motor FIM gain (*p* = 0.0301) and total FIM gain (*p* = 0.0230) using the Mann–Whitney *U*-test. However, the total cognition FIM gain did not differ significantly between groups (*p* = 0.0587) using the Mann–Whitney *U*-test.

### 3.9. Subgroup Analysis

The results of comparing patient characteristics on admission, drug use status, and FIM gain between the polypharmacy- and no polypharmacy-on-discharge groups are shown in Table 12. The polypharmacy-on-discharge group had significantly higher proportion of patients with heart disease (*p* = 0.0026) and had a higher mean number of drugs on admission (*p* < 0.0001) and on discharge (*p* < 0.0001) and ACB score on discharge (*p* = 0.0169) than those in the no polypharmacy-on-discharge group. No significant differences were found in the other variables between the two groups.

## 4. Discussion

After adjusting for differences in baseline variables between groups using PSM, the results showed that a decrease in the number of drugs used among patients with stroke during convalescent rehabilitation was associated with significantly increased gains in motor FIM, cognition FIM, and total FIM compared with those with an increase in the number of drugs used. The findings suggest that reducing, rather than increasing, the number of drugs administered during convalescent rehabilitation could provide better rehabilitation outcomes in patients with stroke. This was especially true in patients taking five or more drugs at the start of rehabilitation. Reducing the overall number of drugs while maintaining the use of medications necessary for secondary prevention is crucial. Therefore, it is important to consider whether the drugs are essential for the patient and proactively revise the drug regimen to ensure rapid rehabilitation of patients with stroke.

Secondary prevention by means of administering antiplatelet agents and anticoagulants and treating risk factors such as hypertension, dyslipidemia, and atrial fibrillation is crucial for preventing recurrent strokes. Lowering blood pressure is essential, with studies suggesting no lower limit to its benefits. The reduction of blood pressure is more important than the specific class of medications used [59, 60]. For dyslipidemia, high-dose atorvastatin, an HMG-CoA reductase inhibitor (statin), reduces the risk of recurrent stroke [61]. Lowering blood pressure is also crucial for secondary prevention of intracerebral hemorrhage [25, 62].

In this study, after PSM, there were no differences between the between the increased and decreased drug use groups in the proportion of patients with a medical history of risk factors for stroke recurrence. Additionally, there were no differences between the two groups in the use of medications for stroke secondary prevention, such as anticoagulants, antiplatelet agents, statins, antidiabetic agents, and antihypertensive drugs. These results suggest that, with careful monitoring for side effects that could hinder rehabilitation, it may be possible to continue using these medications during rehabilitation.

Symptomatic treatment may be provided for various stroke sequelae. Rehabilitation is the main treatment for physical sequelae such as neuroparalysis and mental sequelae such as poststroke depression. Drug therapy is often used alongside rehabilitation to manage these symptoms. Antidepressants such as SSRIs and TCAs are commonly used to alleviate depressive symptoms that hinder rehabilitation progress. However, increased use of SSRIs and TCAs in older adults can increase the risk of falls [63]. Anxiety occurs in 47% of patients during the first year after stroke, and antianxiety medications such as BDZs may be prescribed [64].

In this study, no differences were observed between the increased and decreased groups after PSM in the use of medications for treating sequelae such as poststroke depression, poststroke sleep disorders, and anxiety, such as TCAs, SSRIs, and BDZs, during the ward stay, nor in the ACB scores on admission. However, on discharge, the mean ACB score and the proportion of patients using ACB score 3 drugs were significantly higher in the increased drug use group. Many antidepressants and anxiolytics have anticholinergic effects, and numerous reports highlight the effect of anticholinergic drugs on physical and cognitive functions [65–68]. Our results suggest that the addition of anticholinergic drugs may affect rehabilitation outcomes and support these reports. However, no significant differences were observed between the increased and decreased drug use groups in the use of all drugs related to the ACB score on admission and discharge (data not shown), despite an increase in patients using ACB score 3 drugs in the increased drug use group. Further research is needed to determine whether the cumulative use of anticholinergic drugs affects rehabilitation or whether the effect is due to specific medications.

In the unadjusted analysis comparing all stroke patients based on the presence or absence of polypharmacy on admission, the polypharmacy-on-admission group had a significantly higher proportion of patients aged 65 years and older, higher ACB scores, and a higher proportion of patients with a history of chronic medical conditions and using drugs to treat these conditions. Postacute rehabilitation was also less effective in these patients. These results are consistent with those of previous reports [49, 50, 65–68]. In the comparison of medication changes among three groups of patients based on comparing the number of drugs taken on admission and discharge, the no change group consistently had the highest total FIM scores on admission and on discharge. However, FIM total gain scores did not differ significantly among the three groups. Although the total FIM gain scores were significantly higher in the decreased drug use group than the increased drug use group after PSM, the difference in the proportion of patients exceeding the MCID did not differ significantly between the two groups. Only 23 patients taking five or more drugs on admission were taking fewer than five drugs on discharge. Within the decreased drug use group, there was no significant difference in rehabilitation outcomes between those who discontinued polypharmacy and those who did not.

These results suggest that the relationship between reducing polypharmacy and rehabilitation outcomes is complex. The extent to which medications should be reduced during rehabilitation and whether polypharmacy should be discontinued remain subjects of debate. Further research with larger sample sizes is needed to examine factors that affect rehabilitation and medication use in more detail.

This study has several limitations. First, it was a single-center, retrospective study that did not consider drug dose and administration period. Second, the small sample size likely contributed to the lack of statistically significant differences in cognition FIM total gain in the sensitivity analysis due to low statistical power. Larger sample sizes are needed for future studies. Third, patients without changes in the number of drugs used were excluded due to the two-group PSM design, potentially introducing selection bias. Therefore, caution is needed when interpreting the results, as generalizability may be limited. Additionally, FIM, one of the PBMs, does not cover all aspects such as quality of life and depression, which are reported by patients in PROMs, and other core measures recommended by StrokEDGE II. Therefore, more detailed studies using these outcomes are needed to further investigate the effect of polypharmacy on rehabilitation outcomes of patients with stroke.

This study also has some strengths. It was conducted at a rural hospital, and the patients had a wide age range. Moreover, PSM was performed to effectively balance the baseline variables between the increased and decreased drug use groups. The baseline patient characteristics did not differ between the groups, suggesting that PSM effectively adjusted for confounding by factors other than medication changes.

## 5. Conclusions

In patients with stroke, reducing rather than increasing the number of drugs administered during convalescent rehabilitation could provide better rehabilitation outcomes. It is important to assess the necessity of each medication and proactively revise prescriptions to ensure rapid rehabilitation. Although secondary prevention and medications for stroke sequelae are crucial, careful monitoring of the effectiveness and side effects is essential for effective rehabilitation. Although reducing polypharmacy during stroke rehabilitation is potentially beneficial, further research is needed to determine the optimal extent of medication reduction and discontinuation to enhance rehabilitation strategies.

## Figures and Tables

**Table 1:** Comparison of baseline patient characteristics on admission and drugs used during the stay between the no polypharmacy- and polypharmacy-on-admission groups.

**Patient information**	**No polypharmacy on admission**		**Polypharmacy on admission**		**p**
	**N** = 301		**N** = 354		
	**Number or mean**	**(%) or SD**	**Number or mean**	**(%) or SD**	
Patient characteristics					
Sex (female)	140	(46.5)	172	(48.6)	0.5960
Age (years)⁣^∗^	69.4	13.6	72.9	12.3	0.0007
≥ 65 years⁣^∗^	200	(66.4)	283	(79.9)	< 0.0001
BMI (kg/m^2^) on admission	22.1	3.9	21.8	4.0	0.4942
< 20 kg/m^2^ on admission	98	(32.6)	126	(35.6)	0.4145
Drug use status on admission					
Number of drugs used⁣^∗^	2.6	1.3	7.6	2.5	< 0.0001
ACB score⁣^∗^	0.2	0.5	0.6	1.2	< 0.0001
Score 3 drugs used⁣^∗^	1	(0.3)	20	(5.7)	0.0001
Laboratory values on admission					
High ALT⁣^∗^	79	(26.2)	61	(17.2)	0.0050
eGFR (< 60 mL/min/1.73 m^2^)⁣^∗^	64	(21.3)	124	(35)	0.0001
Alb (< 3.5 g/dL)	85	(28.2)	117	(33.1)	0.1839
Low Hb⁣^∗^	121	(40.2)	192	(54.2)	0.0003
Medical history					
Hypertension	220	(73.1)	281	(79.4)	0.0586
Type 2 diabetes mellitus⁣^∗^	36	(12.0)	137	(38.7)	< 0.0001
Heart disease⁣^∗^	40	(13.3)	86	(24.3)	0.0004
Atrial fibrillation	57	(18.9)	73	(20.6)	0.5901
Dyslipidemia	49	(16.3)	73	(20.6)	0.1548
Hypercholesterolemia	30	(10.0)	45	(12.7)	0.2715
Cerebral infarction⁣^∗^	192	(63.8)	294	(83.1)	< 0.0001
Intracerebral hemorrhage⁣^∗^	105	(34.9)	62	(17.5)	< 0.0001
Subarachnoid hemorrhage	22	(7.3)	15	(4.2)	0.0897
Drugs used during the stay					
Anticoagulants	57	(18.9)	88	(24.9)	0.0689
Antiplatelet agents⁣^∗^	91	(30.2)	184	(52)	< 0.0001
Statins⁣^∗^	55	(18.3)	140	(39.5)	< 0.0001
Antidiabetic agents⁣^∗^	24	(8)	114	(32.2)	< 0.0001
Antihypertensive drugs⁣^∗^	161	(53.5)	234	(66.1)	0.0010
BDZs⁣^∗^	61	(20.3)	118	(33.3)	0.0002
TCAs	3	(1.0)	7	(2.0)	0.3076
SSRIs	2	(0.7)	6	(1.7)	0.2315

*Note:* Welch's *t*-test, mean and standard deviation; chi-square test, number (percentage).

Abbreviations: ACB, anticholinergic cognitive burden; Alb, albumin; ALT, alanine aminotransferase; BDZ, benzodiazepine; BMI, body mass index; eGFR, estimated glomerular filtration rate; Hb, hemoglobin; SD, standard deviation; SSRI, selective serotonin reuptake inhibitor; TCA, tricyclic antidepressant.

⁣^∗^There was a statistically significant difference between the two groups (*p* < 0.05).

**Table 2:** Comparison of FIM total scores between the no polypharmacy- and polypharmacy-on-admission groups.

**FIM total scores**	**No polypharmacy on admission**		**Polypharmacy on admission**		**p**
	**N** = 301		**N** = 354		
	**Number or mean**	**(%) or SD**	**Number or mean**	**(%) or SD**	
On admission					
Motor FIM total	50.6	22.4	51.7	20.2	0.5136
Cognition FIM total	23.4	8.9	23.3	8.3	0.9826
Total FIM	74.0	29.1	75.1	26	0.6186
On discharge					
Motor FIM total	68.1	24.0	66.2	21.6	0.2849
Cognition FIM total	26.3	8.3	25.8	7.9	0.3754
Total FIM	94.5	30.9	92	27.4	0.2791
FIM gain					
Motor FIM total⁣^∗^	17.3	13.4	14.7	12.8	0.0100
≥ 17 (MCID)	143	(47.8)	150	(42.6)	0.1828
Cognition FIM total	2.9	4.1	2.5	3.8	0.1669
≥ 3 (MCID)	126	(42.1)	133	(37.8)	0.2578
Total FIM⁣^∗^	20.2	15.2	17.1	14.7	0.0087
≥ 22 (MCID)	123	(41.1)	123	(34.9)	0.1043

*Note:* Welch's t-test, mean and standard deviation; chi-square test, number (percentage).

Abbreviations: FIM, functional independence measure; MCID, minimally clinically important difference; SD, standard deviation.

⁣^∗^There was a statistically significant difference between the two groups (*p* < 0.05).

**Table 3:** Comparison of baseline patient information on admission between the no change, increased, and decreased drug use groups.

**Patient information on admission**	**No change**		**Increased**		**Decreased**		**p**
	**N** = 128		**N** = 113		**N** = 113		
	**Number or mean**	**(%) or SD**	**Number or mean**	**(%) or SD**	**Number or mean**	**(%) or SD**	
Patient characteristics							
Sex (female)	64	(50)	51	(45.1)	57	(50.4)	0.6711
Age (years)⁣^∗^	71.0	13.3	75.1	10.4	72.8	12.7	0.0256
≥ 65 years⁣^∗^	90	(70.3)	103	(91.2)	90	(79.6)	0.0003
BMI (kg/m^2^)	22.0	4.1	21.9	3.8	21.6	4.3	0.7525
< 20 kg/m^2^	43	(33.6)	39	(34.5)	44	(38.9)	0.6596
Drug use status							
Number of drugs used⁣^∗^	7.2	2.2	7.4	2.6	8.4	2.7	0.0016
ACB score	0.6	1.2	0.7	1.3	0.6	1.0	0.5939
Score 3 drugs used	6	(4.7)	10	(8.9)	4	(3.5)	0.1886
Patients with abnormal laboratory values							
High ALT	20	(15.6)	22	(19.5)	19	(16.8)	0.7254
eGFR (< 60 mL/min/1.73 m^2^)	49	(38.3)	40	(35.4)	35	(31)	0.4921
Alb (< 3.5 g/dL)	40	(31.3)	39	(34.5)	38	(33.6)	0.8548
Low Hb	64	(50)	60	(53.1)	68	(60.2)	0.2737
Medical history							
Hypertension	103	(80.5)	86	(76.1)	92	(81.4)	0.5716
Type 2 diabetes mellitus	50	(39.1)	48	(42.5)	39	(34.5)	0.4672
Heart disease⁣^∗^	20	(15.6)	32	(28.3)	34	(30.1)	0.0159
Atrial fibrillation	22	(17.2)	25	(22.1)	26	(23)	0.4792
Dyslipidemia	23	(18)	27	(23.9)	23	(20.4)	0.5235
Hypercholesterolemia	14	(10.9)	12	(10.6)	19	(16.8)	0.2832
Cerebral infarction⁣^∗^	107	(83.6)	102	(90.3)	85	(75.2)	0.0104
Intracerebral hemorrhage	21	(16.4)	15	(13.3)	26	(23)	0.1440
Subarachnoid hemorrhage	5	(3.9)	2	(1.8)	8	(7.1)	0.1367

*Note:* Welch's *t*-test, mean and standard deviation; chi-square test, number (percentage).

Abbreviations: ACB, anticholinergic cognitive burden; Alb, albumin; ALT, alanine aminotransferase; BMI, body mass index; eGFR, estimated glomerular filtration rate; FIM, functional Independence measure; Hb, hemoglobin; SD, standard deviation.

⁣^∗^There was a statistically significant difference between the three groups (*p* < 0.05).

**Table 4:** Comparison of patient information on discharge and during the stay between the no change, increased, and decreased drug use groups.

**Patient information on discharge or during the stay**	**No change**		**Increased**		**Decreased**		**p**
	**N** = 128		**N** = 113		**N** = 113		
	**Number or mean**	**(%) or SD**	**Number or mean**	**(%) or SD**	**Number or mean**	**(%) or SD**	
Patient characteristics on discharge							
Length of stay (days)	64.6	39.1	74.7	36.7	75.1	39.3	0.0576
BMI (kg/m^2^)	21.7	3.8	21.7	3.5	21.2	3.9	0.5842
BMI < 20 kg/m^2^	47	(37.3)	39	(34.5)	42	(37.2)	0.884
Drug use status on discharge							
Number of drugs used⁣^∗^	7.2	2.2	9.3	2.7	6.7	2.7	< 0.0001
≥ 5 drugs used⁣^∗^	128	(100)	113	(100)	90	(79.6)	< 0.0001
ACB score⁣^∗^	0.8	1.3	1.1	1.5	0.6	1	0.0057
Score 3 drugs used⁣^∗^	9	(7.0)	18	(15.9)	4	(3.5)	0.0030
Patients with abnormal laboratory values during the stay							
High ALT	23	(18)	30	(26.5)	31	(27.4)	0.1572
eGFR (< 60 mL/min/1.73 m^2^)	54	(42.2)	46	(40.7)	40	(35.4)	0.5351
Alb (< 3.5 g/dL)	50	(39.1)	49	(43.4)	46	(40.7)	0.7932
Low Hb⁣^∗^	67	(52.3)	72	(63.7)	80	(70.8)	0.0117
Clinical events during the stay							
Fall	19	(14.8)	19	(16.8)	17	(15)	0.9011
Drugs used during the stay							
Anticoagulants	27	(21.1)	33	(29.2)	28	(24.8)	0.3475
Antiplatelet agents	66	(51.6)	68	(60.2)	50	(44.2)	0.0562
Statins	52	(40.6)	43	(38.1)	45	(39.8)	0.9179
Antidiabetic agents	46	(35.9)	35	(31)	33	(29.2)	0.5062
Antihypertensive drugs	14	(10.9)	12	(10.6)	19	(16.8)	0.2832
BDZs⁣^∗^	30	(23.4)	41	(36.3)	47	(41.6)	0.0084
TCAs	3	(2.3)	2	(1.8)	2	(1.8)	0.9329
SSRIs	2	(1.6)	3	(2.7)	1	(0.9)	0.5818

*Note:* Welch's *t*-test, mean and standard deviation; chi-square test, number (percentage).

Abbreviations: ACB, anticholinergic cognitive burden; Alb, albumin; ALT, alanine aminotransferase; BDZ, benzodiazepine; BMI, body mass index; eGFR, estimated glomerular filtration rate; Hb, hemoglobin; SD, standard deviation; SSRI, selective serotonin reuptake inhibitor; TCA, tricyclic antidepressant.

⁣^∗^There was a statistically significant difference between the three groups (*p* < 0.05).

**Table 5:** Comparison of FIM total scores between the no change, increased, and decreased drug use groups.

**FIM total scores**	**No change**		**Increased**		**Decreased**		**p**
	**N** = 128		**N** = 113		**N** = 113		
	**Number or mean**	**(%) or SD**	**Number or mean**	**(%) or SD**	**Number or mean**	**(%) or SD**	
On admission							
Motor FIM total⁣^∗^	56.3	20.8	48.3	19.0	49.9	19.9	0.0054
Cognition FIM total	24.6	8.1	23.2	8.1	22.1	8.5	0.0711
Total FIM⁣^∗^	80.9	26.4	71.5	24.7	72.1	25.9	0.0072
On discharge							
Motor FIM total⁣^∗^	69.9	21	62.4	23.1	65.9	20.1	0.0349
Cognition FIM total	26.7	7.7	25.4	7.8	25	8.2	0.2133
Total FIM⁣^∗^	96.6	27.1	87.9	28.5	90.9	26.1	0.0470
FIM gain							
Motor FIM total	14.0	13.2	14.1	13	16	12.1	0.4137
≥ 17 (MCID)	51	(40.5)	51	(45.1)	48	(42.5)	0.7674
Cognition FIM total	2.3	3.3	2.3	4.0	2.9	4.0	0.3547
≥ 3 (MCID)	42	(33.3)	42	(37.2)	49	(43.4)	0.2758
Total FIM	16.3	14.6	16.4	15.5	18.9	13.9	0.2996
≥ 22 (MCID)	38	(30.2)	46	(40.7)	39	(34.5)	0.2311

*Note:* Welch's *t*-test, mean and standard deviation; chi-square test, number (percentage).

Abbreviations: FIM, functional independence measure; MCID, minimally clinically important difference; SD, standard deviation.

⁣^∗^There was a statistically significant difference between the three groups (*p* < 0.05).

**Table 6:** Results of the single regression analysis of covariates before propensity score matching.

**Before PSM**	**Increased**			**Decreased**			**p** ** value**	**Std. Dif.**
**Selected covariates**	**Total**	**Number**	**(%)**	**Total**	**Number**	**(%)**		
Cerebral infarction^a^	113	102	(90.3)	113	85	(75.2)	0.0028	0.4063
Heart disease^a^	113	32	(28.3)	113	34	(30.1)	0.7698	0.0389
Type 2 diabetes mellitus^a^	113	48	(42.5)	113	39	(34.5)	0.2186	0.1642
Alb < 3.5 g/dL on admission^a^	113	39	(34.5)	113	38	(38.9)	0.8884	0.0187
BMI < 20.0 kg/m^2^ on admission^a^	113	39	(34.5)	113	44	(33.6)	0.4902	0.0919
Low Hb on admission^a^	113	60	(53.1)	113	68	(60.2)	0.2829	0.1432
	**Total**	**Mean**	**SD**	**Total**	**Mean**	**SD**		
Age (years)^b^	113	75.1	10.4	113	72.8	12.7	0.1367	0.1987
Number of drugs used on admission^b^	113	7.4	2.6	113	8.4	2.7	0.0012	0.3764
Total FIM on admission^b^	113	71.5	24.7	113	72.1	25.9	0.8491	0.0231

Abbreviations: Alb, serum albumin; BMI, body mass index; FIM, functional independence measure; Hb, hemoglobin; PSM, propensity score matching; SD, standard deviation; Std. Dif., standard difference.

^a^Chi-square test.

^b^Welch's *t*-test.

**Table 7:** Results of the single regression analysis of covariates after propensity score matching.

**After PSM**	**Increased**			**Decreased**			**p** ** value**	**Std. Dif.**
**Selected covariates**	**Total**	**Number**	**(%)**	**Total**	**Number**	**(%)**		
Cerebral infarction^a^	78	67	(85.9)	78	66	(84.6)	0.8213	0.0362
Heart disease^a^	78	25	(32.1)	78	23	(29.5)	0.7286	0.0556
Type 2 diabetes mellitus^a^	78	28	(35.9)	78	30	(38.5)	0.7404	0.0531
Alb < 3.5 g/dL on admission^a^	78	27	(34.6)	78	29	(37.2)	0.7385	0.0535
BMI < 20.0 kg/m^2^ on admission^a^	78	33	(42.3)	78	31	(39.7)	0.7448	0.0521
Low Hb on admission^a^	78	51	(65.4)	78	48	(61.5)	0.6179	0.0799
	Total	Mean	SD	Total	Mean	SD		
Age (years)^b^	78	73.7	10.7	78	74.3	12.2	0.7277	0.0559
Number of drugs used on admission^b^	78	7.9	2.8	78	8.1	2.6	0.6376	0.0756
Total FIM on admission^b^	78	71.5	26.2	78	70.2	25.6	0.6045	0.0831

Abbreviations: Alb, serum albumin; BMI, body mass index; FIM, functional independence measure; Hb, hemoglobin; PSM, propensity score matching; SD, standard deviation; Std. Dif., standard difference.

^a^Chi-square test.

^b^Welch's *t*-test.

**Table 8:** Comparison of patient characteristics on admission in the increased and decreased drug use groups after propensity score matching.

**Patient information on admission**	**Increased**		**Decreased**		**p**
	**N** = 78		**N** = 78		
	**Number or mean**	**(%) or SD**	**Number or mean**	**(%) or SD**	
Patient characteristics					
Sex (female)	32	(41)	36	(46.2)	0.5184
Age (years)	73.7	10.7	74.3	12.2	0.7277
Age ≥ 65 years	69	(88.5)	66	(84.6)	0.4816
BMI (kg/m^2^)	21.1	3.6	21.4	4.3	0.6038
BMI < 20 kg/m^2^	33	(42.3)	31	(39.7)	0.7448
Patients with abnormal laboratory values					
High ALT	11	(14.1)	14	(17.9)	0.5126
eGFR (< 60 mL/min/1.73 m^2^)	29	(37.2)	25	(32.1)	0.5008
Alb (< 3.5 g/dL)	27	(34.6)	29	(37.2)	0.7385
Low Hb	55	(70.5)	57	(73.1)	0.7220
Medical history					
Hypertension	60	(76.9)	62	(79.5)	0.6981
Type 2 diabetes mellitus	28	(35.9)	30	(38.5)	0.7404
Heart disease	25	(32.1)	23	(29.5)	0.7286
Atrial fibrillation	18	(23.1)	21	(26.9)	0.5791
Dyslipidemia	18	(23.1)	13	(16.7)	0.3158
Hypercholesterolemia	9	(11.5)	13	(16.7)	0.3575
Cerebral infarction	67	(85.9)	66	(84.6)	0.8213
Intracerebral hemorrhage	14	(17.9)	13	(16.7)	0.8324
Subarachnoid hemorrhage	1	(1.3)	5	(6.4)	0.0958

*Note:* Welch's *t*-test, mean and standard deviation; chi-square test, number (percentage). The two groups had no significant differences (*p* < 0.05).

Abbreviations: Alb, albumin; ALT, alanine aminotransferase; BMI, body mass index; eGFR, estimated glomerular filtration rate; Hb, hemoglobin; SD, standard deviation.

**Table 9:** Comparison of patient information on discharge and during the stay after propensity score matching.

**Patient information on discharge or during the stay**	**Increased**		**Decreased**		**p**
	**N** = 78		**N** = 78		
	**Number or mean**	**(%) or SD**	**Number or mean**	**(%) or SD**	
Patient characteristics on discharge					
Length of stay (days)	69.6	34.6	75.2	37.5	0.3328
BMI (kg/m^2^)	21.1	3.3	21.0	4.0	0.8739
BMI < 20 kg/m^2^	31	(39.7)	28	(35.9)	0.6204
Patients with abnormal laboratory values during the stay					
High ALT	18	(23.1)	23	(29.5)	0.3631
eGFR (< 60 mL/min/1.73 m^2^)	34	(43.6)	29	(37.2)	0.4146
Alb (< 3.5 g/dL)	33	(42.3)	37	(47.4)	0.5196
Low Hb	55	(70.5)	57	(73.1)	0.7220
Clinical events during the stay					
Fall	13	(16.7)	10	(12.8)	0.4981

*Note:* Welch's *t*-test, mean and standard deviation; chi-square test, number (percentage). The two groups had no significant differences (*p* < 0.05).

Abbreviations: Alb, albumin; ALT, alanine aminotransferase; BMI, body mass index; eGFR, estimated glomerular filtration rate; Hb, hemoglobin; SD, standard deviation.

**Table 10:** Comparison of drug use status on admission and discharge after propensity score matching.

**Drug use status**	**Increased**		**Decreased**		**p**
	**N** = 78		**N** = 78		
	**Number or mean**	**(%) or SD**	**Number or mean**	**(%) or SD**	
Number of drugs used					
On admission	7.9	2.8	8.1	2.6	0.6376
On discharge⁣^∗^	9.7	3.0	6.5	2.5	< 0.0001
Patients with polypharmacy					
On admission	78	(100)	78	(100)	−
On discharge⁣^∗^	78	(100)	60	(76.9)	< 0.0001
ACB score					
On admission	0.8	1.5	0.6	1.0	0.2031
Score 3 drugs used⁣^∗^	8	(10.3)	2	(2.6)	0.0498
On discharge⁣^∗^	1.2	1.7	0.5	0.8	0.0004
Score 3 drugs used⁣^∗^	14	(17.9)	1	(1.3)	0.0004
Drugs used during the stay					
Anticoagulants	23	(29.5)	20	(25.6)	0.5909
Antiplatelet agents	45	(57.7)	34	(43.6)	0.0781
Statins	27	(34.6)	25	(32.1)	0.7341
Antidiabetic agents	20	(25.6)	25	(32.1)	0.3769
Antihypertensive drugs	48	(61.5)	49	(62.8)	0.8689
BDZs	31	(39.7)	32	(41)	0.8704
TCAs	1	(1.3)	2	(2.6)	0.5599
SSRIs	3	(3.8)	1	(1.3)	0.3110

*Note:* Welch's *t*-test, mean and standard deviation; chi-square test, number (percentage).

Abbreviations: ACB, anticholinergic cognitive burden; BDZ, benzodiazepine; CCB, calcium channel blocker; SSRI, selective serotonin reuptake inhibitor; TCA, tricyclic antidepressant.

⁣^∗^The number of drugs used on discharge (*p* < 0.0001), the proportion of patients with polypharmacy on discharge (*p* < 0.0001), and the ACB score on discharge (*p* = 0.0004) were significantly lower (*p* < 0.05) in the decreased drug use group than in the increased drug use group.

**Table 11:** FIM total scores on admission and discharge and FIM gain in the increased and decreased drug use groups after propensity score matching.

**FIM total scores**	**Increased**		**Decreased**		**p**
	**N** = 78		**N** = 78		
	**Number or mean**	**(%) or SD**	**Number or mean**	**(%) or SD**	
On admission					
Motor FIM total	48.9	20.0	48.5	19.9	0.8981
Cognition FIM total	23.4	8.2	21.6	8.6	0.1965
Total FIM	72.3	26.2	70.2	25.6	0.6045
On discharge					
Motor FIM total	61.4	24.1	66.1	19.3	0.1829
Cognition FIM total	25.4	8.2	25.1	8.2	0.8143
Total FIM	86.8	30.5	91.2	24.9	0.3285
FIM total gain					
Motor FIM total⁣^∗^	12.5	13.7	17.6	11.8	0.0139
≥ 17 (MCID)	31	(39.7)	41	(52.6)	0.1083
Cognition FIM total⁣^∗^	2.0	4.2	3.4	4.4	0.0377
≥ 3 (MCID)	28	(35.9)	37	(47.4)	0.1439
Total FIM⁣^∗^	14.5	16.3	21.0	13.8	0.0077
≥ 22 (MCID)	29	(37.2)	32	(41.0)	0.6226

*Note:* Welch's *t*-test, mean and standard deviation (SD); chi-square test, number (percentage).

Abbreviation: FIM, functional independence measure; MCID, minimally clinically important difference.

⁣^∗^The total motor FIM (*p* = 0.0139), total cognition FIM (*p* = 0.0377), and total FIM (*p* = 0.0077) were significantly higher (*p* < 0.05) in the decreased group than in the increased group.

**Table 12:** Comparison of patient characteristics on admission, drug use status, and FIM gain in the polypharmacy- and no polypharmacy-on-discharge subgroups of the decreased drug use group.

	**Polypharmacy on discharge**		**No polypharmacy on discharge**		**p**
	**N** = 90		**N** = 23		
	**Number or mean**	**(%) or SD**	**Number or mean**	**(%) or SD**	
Patient characteristics on admission					
Patient information					
Sex (female)	48	(53.3)	9	(39.1)	0.2241
Age (year)	74	12.1	68	14	0.0681
Age ≥ 65 years	74	(82.2)	16	(69.6)	0.1785
Medical history					
Hypertension	75	(83.3)	17	(73.9)	0.3000
Type 2 diabetes mellitus	35	(38.9)	4	(17.4)	0.0529
Heart disease⁣^∗^	33	(36.7)	1	(4.3)	0.0026
Atrial fibrillation	22	(24.4)	4	(17.4)	0.4732
Dyslipidemia	20	(22.2)	3	(13)	0.3292
Hypercholesterolemia	17	(18.9)	2	(8.7)	0.2434
Cerebral infarction	70	(77.8)	15	(65.2)	0.2131
Intracerebral hemorrhage	18	(20)	8	(34.8)	0.1328
Subarachnoid hemorrhage	6	(6.7)	2	(8.7)	0.7349
Drug use status					
Number of drugs used					
On admission⁣^∗^	9.1	2.6	5.6	0.8	< 0.0001
On discharge⁣^∗^	7.6	2.4	3.5	0.9	< 0.0001
ACB score					
On admission	0.6	1.1	0.4	0.9	0.4532
Score 3 drugs used	4	(4.4)	0	(0)	0.3033
On discharge⁣^∗^	0.6	1	0.3	0.5	0.0169
Score 3 drugs used	4	(4.4)	0	(0)	0.3033
FIM gain					
Motor FIM total	15.8	12	16.7	12.6	0.7663
≥ 17 (MCID)	39	(43.3)	9	(39.1)	0.7159
Cognition FIM total	2.9	4.4	3	2.3	0.8546
≥ 3 (MCID)	35	(38.9)	14	(60.9)	0.0576
Total FIM	18.7	14.1	19.7	13	0.7497
≥ 22 (MCID)	32	(35.6)	7	(30.4)	0.6448

*Note:* Welch's *t*-test, mean and standard deviation; chi-square test, number (percentage).

Abbreviations: ACB, anticholinergic cognitive burden; BMI, body mass index; FIM, functional independence measure; MCID, minimally clinically important difference; SD, standard deviation.

⁣^∗^The proportion of patients with heart disease (*p* = 0.0026), the number of drugs used on admission (*p* < 0.0001) and on discharge (*p* < 0.0001), and ACB score on discharge (*p* = 0.0169) were significantly higher in the polypharmacy-on-discharge group than in the no polypharmacy-on-discharge group.

## Data Availability

The study dataset cannot be publicly shared to protect the privacy of individuals who participated in the study. The data will be shared upon reasonable request to the corresponding author.
